# Supplementary material for: Analysis of chi angle distributions in free amino acids via multiplet fitting of proton scalar couplings
Source: Magn Reson (Gott). 2024 Aug 19;5(2):103–20. doi: 10.5194/mr-5-103-2024 (PMC11570886; doi:10.5194/mr-5-103-2024)
Supplement: The supplement related to this article is available online at: https://doi.org/10.5194/mr-5-103-2024-supplement. [file mr-5-103-2024-supplement.zip › Syed2024/data/fit1d_fitnmr_output.tar.gz!/fit1d/gln/400/bmse000038_simulation_1_nmredata/1H/sparse_1d.pdf]

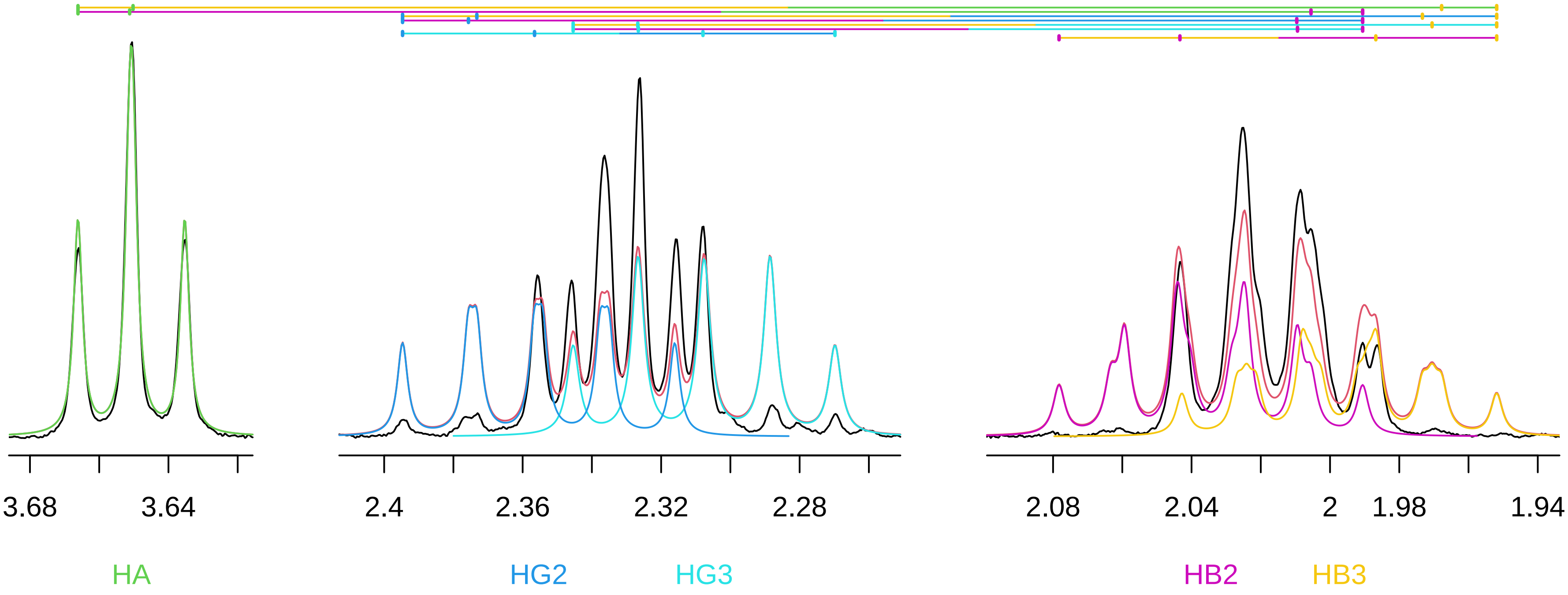
3.68
3.64
2.4
2.36
2.32
2.28
2.08
2.04
2
1.98
1.94
HA
HG2
HG3
HB2
HB3
